# An empirical approach to the “Trump Effect” on US financial markets with causal-impact Bayesian analysis

**DOI:** 10.1016/j.heliyon.2020.e04760

**Published:** 2020-08-26

**Authors:** Pedro Antonio Martín Cervantes, Salvador Cruz Rambaud

**Affiliations:** Departamento de Economía y Empresa, Universidad de Almería, Spain

**Keywords:** Causality, Causal-impact Bayesian analysis, Efficient market hypothesis, Market anomalies, Calendar effect, Economics, Finance, Behavioral economics, Econometrics, Business

## Abstract

In this paper, we have tested the existence of a causal relationship between the arrival of the 45th presidency of United States and the performance of American stock markets by using a relatively novel methodology, namely the causal-impact Bayesian approach. In effect, we have found strong causal relationships which, in addition to satisfying the classical Granger Causality linear test, have been quantified in absolute and relative terms. Our findings should be included in the context of one of the main markets anomalies, the so-called “calendar effects”. More specifically, when distinguishing between the subperiods of pre- and post-intervention, data confirm that the “US presidential cycle” represents a process of high uncertainty and volatility in which the behavior of the prices of financial assets refutes the Efficient-Market Hypothesis.

## 1 Introduction

The idea of linking political events to the performance of financial assets is quite old: it can already be found in Penso de la Vega (1688), which can be historically considered as the first work in analyzing the behavior of financial markets. This paper describes a series of alternative and unpredictable guidelines, assimilable to the current conceptualization of *bull* and *bear* markets which, according to Goetzmann (1993), have been consecutively repeated over the last three hundred years.

These generic patterns are clearly linked to certain environmental factors such as political changes which usually give rise to behavioral biases in the process of decision-making by investors, which should be analyzed by taking into account the transcendental importance of psychological factors in financial decision-making (see, e.g., Muradoglu and Harvey, 2012). Among these effects, we should highlight the “social moods” (Prechter et al., 2012), “the noise” in the sense of Black (1986), in a context in which the impact of political institutions plays an essential rôle in corporate risk-taking (Boubakri et al., 2013) and the “calendar effects” (Urquhart and McGroarty, 2014) which is any market anomaly involving observed unusual or abnormal return on investments around certain dates or periodic events.

Obviously, accepting the existence of calendar effects in a given financial market presupposes the complete rejection of the rational investment paradigm of the Efficient Market Hypothesis (hereinafter EMH, see Fama, 1965, Fama, 1970). However, in the opinion of Lo (2012), this conceptual framework “is not wrong; it is merely incomplete”. In principle, the calendar effects could be analyzed by using any of the three main hypotheses which refute the EMH. Taking into account the information available to investors, the Overreaction Hypothesis (hereinafter OH, see DeBondt and Thaler, 1985, DeBondt and Thaler, 1987) states that investors overreact to positive or negative news which is the key element for upward or downward movements of financial assets. The Uncertain Information Hypothesis (hereinafter UIH, see Brown et al., 1988, Brown et al., 1993, Bouoiyour and Selmi, 2017) establishes that the level of uncertainty and risk in the financial markets will increase with information which is unexpected. Therefore, investors are unable to react to unexpected information shocks, and tend to undervalue the financial assets.

Finally, the Adaptive Market Hypothesis (hereinafter AMH, see Lo and MacKinlay, 1988, Lo, 2004, Lo, 2005) harmonizes the investors' rationality and the behavioral biases in an evolutive framework. More specifically, Lo (2005) summarizes the conceptual basis of this hypothesis as follows: “based on evolutionary principles, the adaptive market hypothesis implies that the degree of market efficiency is related to environmental factors characterizing market ecologies such as the number of competitors in the market, the magnitude of profit opportunities available, and the adaptability of the market participants”.

Needless to say that the AMH is the most widespread hypothesis in the existing literature as it points out the empirical inconsistencies of the EMH derived from several tests such as the Automatic Variance Ratio test (Lo and MacKinlay, 1988), the Automatic Portmanteau test (Kim et al., 2011), the Generalized Spectral test (Kim et al., 2011), etc. However, the OH and the UIH can also help to explain these.

Following an approach similar to Born et al. (2017), who employed a single-index regression model without specifically adhering to any of the hypotheses contrary to the EMH, this research work has opted to implement a causal methodology based on structural equations (Bańbura et al., 2011). Specifically, a causal analysis of the so-called “Trump Effect” has been carried out through a procedure which, to the extent of our knowledge, has rarely been employed in the financial field: the causal-impact Bayesian analysis (Brodersen et al., 2015). Using this specific methodology, it is not only possible to infer whether there exists a causal relationship between two variables *x* and *y*, but also to quantify and predict this impact from a cut-off point *t* or “intervention” where the incidence of a given event appears. Consequently, this paper represents *prima facie* a completely new application of this methodological corpus in the economic and financial field, when analyzing the impact of arrival of the Trump Administration on the US financial markets by using several criteria such as its interpretation, prediction, tendency or contrast.

In order to achieve the objectives proposed in this research, this paper's structure continues as follows: Section 2 presents a state of the art of the econometric analysis of the “Trump Effect”, contextualized in the so-called “Presidential Election Cycle”. Next, Section 3 describes and justifies the material and methods employed. Section 4 gives the achieved results which are discussed and put into the literature perspective in Section 5. Finally, Section 6 renders the main conclusions obtained from this work and also outlines an investment strategy based on the use of the Brodersen et al. (2015) procedure which, in an immediate future, may be of particular interest to investors and practitioners.

## 2 Literature review

In spite of the fact that each institutionalized market presents its own specific characteristics before the advent of any political event considered transcendent (Erb et al., 1996), in the American financial markets a specific phenomenology has been described related to the passage from one presidency to another, empirically quantified in the Stock Trader's Almanac of Y. Hirsch, one of the reference manuals for US investors (Hirsch and Hirsch, 2010). According to Valadez and Nickles (2013), the main patterns defining this effect can be included in a “Presidential Election Cycle” theory, a phenomenon which, in the opinion of Sturm (2016), not only transcends the financial markets, but also affects the valuation of the main accounting ratios of US companies.

For Allvine and O'Neill (1980), the empirical unsustainability of the EMH is endorsed by the “Presidential Election Cycle”, given that according to this study, during certain phases of the cycle it is feasible to implement a “buy and hold” strategy able to generate an almost constant return rate, by rejecting the randomness of stock market prices and, obviously, their supposed unpredictability.

Other early works which examined the influence of a change of president on the dynamics of US financial markets (see, e.g., Niederhoffer et al., 1970, Forsythe et al., 1992) focused on determining their most representative characteristics, such as the uncertainty, the presence of speculative movements, or the indecision of the main financial interlocutors, in the face of the advent of a political change whose final outcome is uncertain. Santa-Clara and Valkanov (2003) pointed out the complexity of the phenomenon analyzed by stating that the difference in returns throughout the political cycle can be considered as a complex “puzzle”. Wong and McAleer (2009) tried to clarify this confusion by using a spectral analysis and an EGARCH intervention model to infer that, in every presidency, since 1965, the US stock market has exhibited certain quantitative patterns which empirically support the existence of a “Presidential Election Cycle”. For their part, Prechter et al. (2012) used a typically behavioral perspective, namely the “social moods”, to find a positive and relatively strong relationship between the margin of votes cast for one presidential candidate and the previous net percentage change in the US stock market.

With respect to the incidence of the most recent presidency of the United States on the financial markets, several studies have analyzed in depth a series of peculiarities derived from its personal character which Nikiforos (2017) labeled as “Trump Effect”. A large part of these works, including Benton and Philips (2019) and Brans and Scholtens (2020), have been focused on reflecting quantitatively the impact of Trump's preferred public communication tool, viz Twitter (Vlatković, 2018), on some *ad hoc* newly created indices, such as the Volfefe index (Klaus and Koser, 2020), a stock market index of volatility based on generalized market attitudes towards US Treasury bonds, linked to the current US President's tweets (see, e.g., Alloway, 2019).

The old adage “When America sneezes, the world catches a cold”, so often evidenced in geopolitics and international finance (see, e.g., Suardi, 2010), is readily apparent when analyzing the “Trump Effect”. In fact, its impact has been studied both exclusively in US stock exchanges (Born et al., 2017, Wagner et al., 2017, Wagner et al., 2018a, Wagner et al., 2018b, Cox and Griffith, 2019) and in other stock exchanges as the European (Klaus and Koser, 2020) as well as the Mexican, Japanese, Australian and Brazilian markets (de Area Leão Pereira et al., 2018).

A common denominator of these researches is the fact of finding a more or less direct relationship, even during the election campaign, between the almost daily flow of Trump's tweets (positives/negatives) (or Google Trends impact) and the performance of US financial markets and foreign exchange markets. In effect, according to Colonescu (2018), there exists a significant short-term effect of tweets on the USD/Canadian Dollar exchange rate, practically analogous to that described by Benton and Philips (2019) in the USD/Mexican Peso exchange rate.

One of the stylized facts which characterizes the “Trump Effect”, viz the uncertainty, has been studied from several stand points. For example, Bouoiyour and Selmi (2017) analyze through the UIH at a sectoral level, the reaction of markets to the continual appearance of unanticipated information from the Trump Administration, by obtaining a very unequal response from different sectors. According to this analysis, in general terms, the effect was positive in some specific sectors or industries (healthcare, oil and gas, real estate, defense, financials and consumer goods and services) and relatively negative in others (mainly technology and utilities). The study of uncertainty has also transcended into the macroeconomic field. In this way, Selmi et al. (2020) underlined that economic and monetary policy-related uncertainties are key factors to explain the observed changes in inflation with the arrival of the 45th presidency of the United States, characterized by a continual increase in volatility.

Targeting the American financial markets, Wagner et al. (2017) suggested that the government change led to a massive adjustment of the differential in the prices of financial assets caused by the uncertainty derived from the different economic measures implemented by the Trump Administration, mainly the Tax Cuts and Jobs Act of 2017 (TCJA), a set of fiscal reforms which would have the short term consequence of a massive arrival of capital to the American stock exchanges. These reforms would collaterally generate, in the opinion of Wagner et al. (2017), a conspicuous increase in speculation, as a result of the volatility of markets which were optimistic about the final dénouement of the economic policies brought by the Trump Administration to the White House (trade, fiscality and labor).

This wave of speculative flows ceased when investors were able to anticipate the cut in corporate taxes as promised in the pre-election campaign (Wagner et al., 2018b), leading to an almost generalized rise in stock prices, due to the reactions of companies in the face of a foreseeable improvement of their profits as per the expectations of shareholders who saw how their transaction costs were reduced. In any case, as a result of the first measures proposed by the Trump Administration, the only doubts of investors would apply to those US companies with high non-US revenues (Wagner et al., 2018a). It should be pointed out that in the socio-economic scenario designed by this administration, any dynamic strategy of diversification into non-American financial assets would be somewhat limited given the almost total prevalence of this country's financial assets in accordance with the motto “America First”. Therefore, the “Trump Effect” indirectly entails a restriction on the quasi-traditional internationalization-diversification of US securities portfolios into other financial markets such as the UK or Japan (Miralles-Marcelo et al., 2015).

## 3 Materials and methods

For the elaboration of this paper, a research protocol inspired in Kallet (2004) has been followed, as the basic framework to be used in the study of the phenomenon to be analyzed, and determines the theoretical framework on which the Bayesian causal-impact analysis is based (Brodersen et al., 2015). Next, the used dataset has been defined, explicitly indicating the reasons for its selection and specifying its nature and time horizon.

### 3.1 Methodology

The predictability of classical econometric procedures based on the Causality of Granger (Granger, 1969) depends on determining certain causal relationships which can change in times of financial turbulence and economic uncertainty, as indeed was the period immediately before the arrival of the Trump Administration (Wagner et al., 2018a, Wagner et al., 2018b). In these contexts, Shi et al. (2018) claim the need to obtain evolutionary econometric models more suitable for frequent and unexpected financial shocks. An optimal procedure respecting these conditions is that proposed by Brodersen et al. (2015), a new type of Bayesian structural time-series model (Scott and Varian, 2014, Scott and Varian, 2015). This approach starts from the classic structural pair-equations model (Scott and Varian, 2014) which links $y_{t}$ to $\alpha_{t}$, a vector of latent state variables:

(1) $$y_{t}=Z_{t}^{T}\alpha_{t}+\varepsilon_{t},\;\varepsilon_{t}∼N(0,\sigma_{t}^{2}),$$

(2) $$\alpha_{t+1}=T_{t}\alpha_{t}+R_{t}\eta_{t},\;\eta_{t}∼N(0,Q_{t}),$$

where $y_{t}$ denotes a scalar observation, $Z_{t}$, a *d*-dimensional output vector, $T_{t}$, a d×d transition matrix, $R_{t}$, a d×q control matrix, $\varepsilon_{t}$, a scalar observation error with noise variance $\sigma_{t}$, and $\eta_{t}$ represents a *q*-dimensional system error with a q×q state-diffusion matrix $Q_{t}$, being q≤d. The error terms $\varepsilon_{t}∼N(0,\sigma_{t}^{2})$ and $\eta_{t}∼N(0,Q_{t})$ are independent of all other unknowns.

Under this generic scheme, Eq. (1) is called the *observation equation*, relating the observed data, $y_{t}$, with an unobserved latent *d*-dimensional state vector $\alpha_{t}$, and Eq. (2) is called the *transition equation* (or *state equation*), which defines how the latent state vector, $\alpha_{t}$, evolves over time (*t*). Since the error structure is defined as $R_{t}\eta_{t}$, this feature allows for the inclusion of different components of the state of less than full rank such as a *local linear trend* and a *seasonality* component.

In the Brodersen et al. (2015) model, the *local linear trend* is determined by the two Eqs. (3), where $\mu_{t}$ denotes the specific value of the trend variable at time *t*, $\delta_{t}$ is the expected increase in *μ* within the temporal window [*t*,t+1], the error terms being $\eta_{\mu ,t}∼N(0,\sigma_{\mu }^{2})$ and $\eta_{\delta ,t}∼N(0,\sigma_{\delta }^{2})$:

(3) $$\begin{array}{cc} & \mu_{t+1}=\mu_{t}+\delta_{t}+\eta_{\mu ,t},\;\eta_{\mu ,t}∼N(0,\sigma_{\mu }^{2}),\\ & \delta_{t+1}=\delta_{t}+\eta_{\delta ,t},\;\eta_{\delta ,t}∼N(0,\sigma_{\delta }^{2}). \end{array}$$

**Figure 1:**
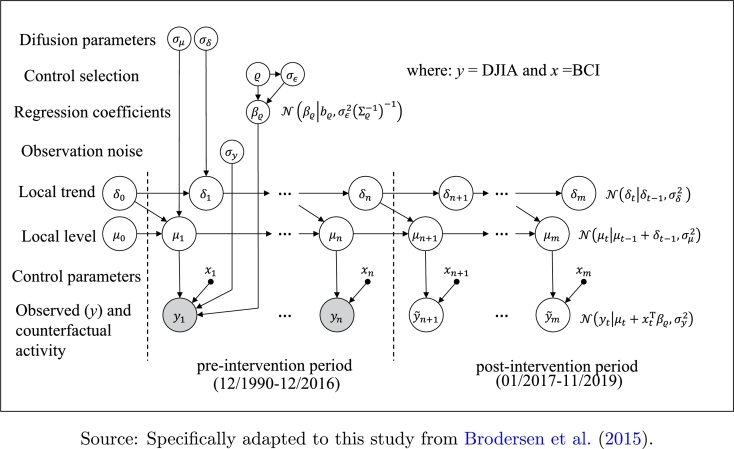
Causal-impact scheme.

In the same way, the *seasonality* factor in the time domain is given by Eq. (4):

(4) $$\gamma_{t+1}=-\sum_{S=0}^{S-2}\gamma_{t-S}+\eta_{\gamma ,t},$$

where *S* denotes the number of seasonal periods and $\gamma_{t}$ represents their joint contribution to the observed response variable ($y_{t}$). The procedure for attaching control series in the model is via a linear regression, whose coefficients can be static (contemporaneous covariates with static coefficients, see Eq. (5)):

(5) $$Z_{t}=\beta^{T}x_{t},\;\text{if }\alpha_{t}=1$$

or time-varying (contemporaneous covariates with dynamic coefficients, see Eq. (6)):

(6) $$\begin{array}{c} x_{t}^{T}\beta_{t}=\sum_{j=1}^{J}x_{j,t}\beta_{j,t},\\ \beta_{j,t+1}=\beta_{j,t}+\eta_{\beta ,j,t}, \end{array}$$

where $\beta_{j,t}$ represents the *j*th control series-coefficient with error terms distribution equal to $\eta_{\beta ,j,t}∼N(0,\sigma_{\beta_{j}}^{2})$, where $\sigma_{\beta_{j}}$ denotes the standard deviation of its linked random walk (which allows the dynamic coefficients calculation). The final assembled state-space model comes from the appropriate selection of components for trend, seasonality and static-dynamic regressions. In Fig. 1 the causal procedure proposed by Brodersen et al. (2015) is outlined by indicating its implementation in this work.

### 3.2 Data description

Our dataset contains monthly data of DJIA-adjusted prices (from Bloomberg database) and the Business Confidence Index (hereinafter BCI, see OECD, 2019). For both variables, the analyzed period ranges from 1991/01 to 2019/11, thus obtaining a total of 696 monthly observations. This index has been selected to measure the “Trump Effect” taking into account that it is a clear indicator of the sentiment of OECD economies, evaluating, among other aspects, the investment activity of each country. In effect, when it is above 100, it denotes an economic context of “optimism”, showing confidence in the most immediate future business performance and, conversely, when it is below 100, it describes a “pessimistic” industrial environment linked to adverse short term future growth perspectives. Following Khoi et al. (2016), the inclusion of this index as a part of the analyzed dataset is justified by taking into account its proven ability to: 1) foresee the perception of investors about future plans on capital investment, 2) analyze the impact of price on demand, and 3) create a general perspective for international investors and multinational enterprises of the business activities of each under consideration country.

In the same way, we have also taken the BCI as a reference for merely operational reasons. As already indicated, other works of a similar nature have used, as a priority, the flow of Google Trends or tweets issued by President Trump. However, as this daily flow lacks continual periodicity, it allows neither the possibility of establishing a time horizon prior to the beginning of Twitter operations (2006) nor do all sources consider it as sufficiently objective (see, e.g., Molyneux et al., 2016). Furthermore, since the methodology used in this work is based on the use of structural equations (Brodersen et al., 2015), we have employed an approach relatively similar to Scott and Varian (2015) by considering the BCI as a more suitable indicator to measure investment activity than the University of Michigan monthly survey of Consumer Sentiment.

**Figure 2:**
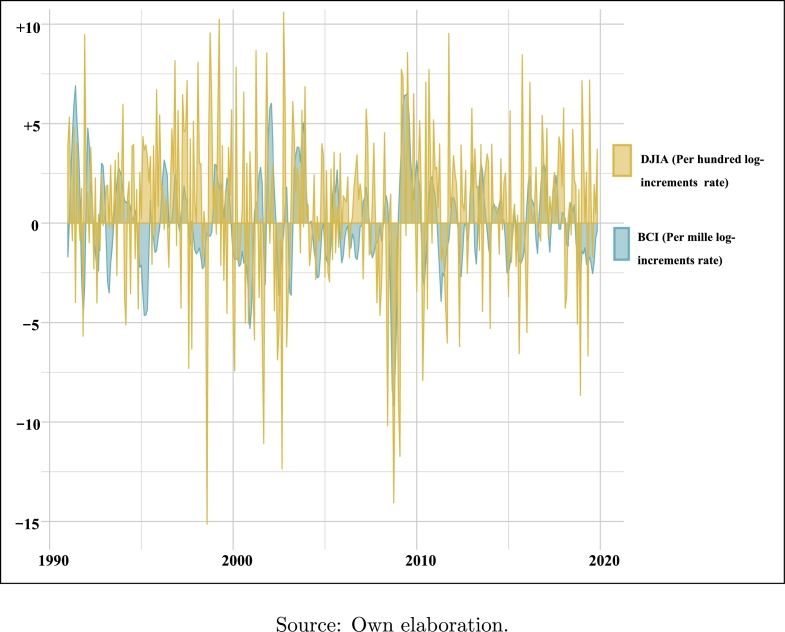
DJIA vs. BCI. Monthly log-increment rates, sample: 1991M01-2019M11.

Fig. 2 exhibits the joint evolution of these variables through the selected time horizon on a log-increments basis. Given that the variability of the DJIA is much greater than the BCI, which would make it impossible to assess adequately the performance of the variables if both are expressed in percentage units, it has been decided to represent variable BCI in per mille units.

Summarized statistics and a difference of means test have been reported in Table 1. In the case of the mean, both variables show positive values: 0.00681 and 0.00004, respectively. The main defining note of the BCI is its limited range, which is logical because it is a relatively stable indicator whose values are always very close to 100. The DJIA exhibits a variability greater than the BCI: in annual terms, the standard deviation of the DJIA is 0.0403 and 0.0023 of the BCI ($\text{annualized st. dev.}=\text{monthly sd. dev.}\times \sqrt{12}$). In neither case, the variables meet the hypotheses of normality, exhibiting an excess of kurtosis. Likewise, a relative symmetry of the BCI variable can be observed, compared to a very slight asymmetry of the DJIA (negative skewness). Similarly, based on the Welch *t*-test (Welch, 1951), carried out on the difference between the two populations analyzed ($\mu_{X}$ vs. $\mu_{Y}$), assuming non-normality and non-equal variances, it can be considered that the means of both populations are significantly different.

**Table 1:** Summary of statistics and difference of means contrast.

I. Summary of statistics					
Statistics (A)	DJIA	BCI	Statistics (B)	DJIA	BCI
Observations	347	347	SE Mean	0.0021	0.00012
Minimum	−0.1640	−0.0092	Variance	0.00162	0.000005
Maximum	0.1007	0.0068	Standard deviation	0.0403	0.0023
Range	0.2648	0.0160	Coefficient of variation	5.9148	49.5585
Sum	2.3656	0.0161	JB *p*-value	<0.01	<0.01
Median	0.0098	−0.00007	Skewness	−0.760081	0.028845
Mean	0.00681	0.00004	Kurtosis	4.698722	4.261965


II. Difference of means contrast			
Two-sample Welch *t*-test (Welch, 1951):			
*μ*_*X*_: population mean of DJIA	Difference: $\mu_{X}-\mu_{Y}$		
*μ*_*Y*_: population mean of BCI	Equal variances are not assumed		
Null hypothesis $H_{0}$: $\mu_{X}-\mu_{Y}=0$	*t*-test	d.f.	*p*-value
Alternative hypothesis $H_{1}$: $\mu_{X}-\mu_{Y}\neq 0$	−35.17	347	0.00

**Figure 3:**
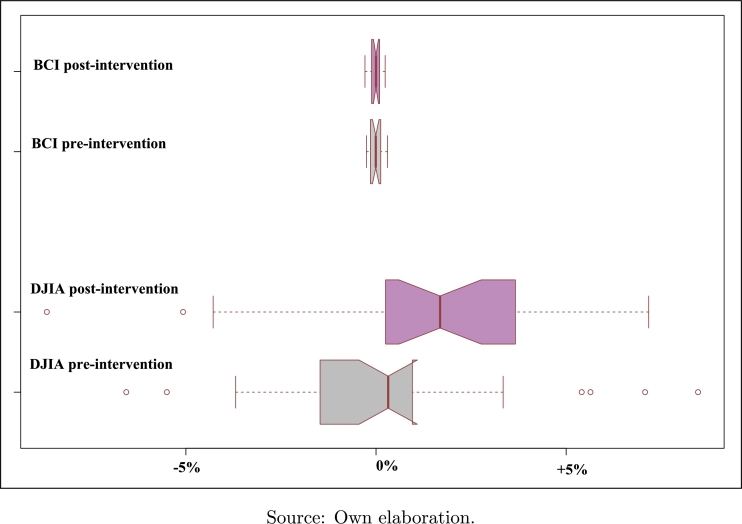
DJIA and BCI boxplots. Pre-intervention and post-intervention periods.

Fig. 3 exhibits a boxplot of the performance of both variables, ±2 years from the beginning of the 45th US Administration. Thus, in this figure, the pre-intervention period is 2015/01-2016/12, and the post-intervention is 02/2017-01/2019. An increment of DJIA can be observed which ranges from 0.493% (before Trump Administration) to 1.026% (after Trump Administration). It could be inferred that this presidential change had positive effects on the American stock markets (DJIA), an observation which could be confirmed in terms of causality according to Table 2, in which Granger's causality test points to an alleged bilateral causal relationship among the variables, given four different lag lengths (K=2,3,4 and 5).

**Table 2:** Pairwise causality test according to Granger (Granger, 1969). Monthly variables DJIA and BCI (in log-terms, sample: 1991M01-2019M11).

Null hypothesis (K=2):	Obs.	F-Statistic	Prob.
BCI does not cause DJIA	345	6.57954	0.0016
DJIA does not cause BCI		1.88663	0.1532


Null hypothesis (K=3):	Obs.	F-Statistic	Prob.
BCI does not cause DJIA	344	4.31576	0.0053
DJIA does not cause BCI		1.01442	0.3864


Null hypothesis (K=4):	Obs.	F-Statistic	Prob.
BCI does not cause DJIA	343	3.21193	0.0132
DJIA does not cause BCI		0.75393	0.5560


Null hypothesis (K=5):	Obs.	F-Statistic	Prob.
BCI does not cause DJIA	342	2.52817	0.0290
DJIA does not cause BCI		1.17713	0.3201

## 4 Empirical results

Once the analysis period has been subdivided into the pre-intervention (01/1991-12/1996) and the post-intervention (01/2017-11/2019) periods, considering the arrival of the Trump Administration as the axis of our causal analysis (intervention = 01/2017) and typifying the DJIA as a measure of the financial market performance and the BCI index as a *proxy* of the investors' level of satisfaction in the United States, the application of the methodology presented by Brodersen et al. (2015) on both variables indicates a significant effect of the arrival of this administration on the performance of US financial markets, as verified in Table 3, following the average and cumulative approaches.

**Table 3:** Summary of the causal impact analysis of “Trump Effect”.

	Average approach	Cumulative approach
Actual	24,381	853,338
Prediction (S.D.)	18,680 (968)	653,795 (33,878)
95% CI	[16,808-20,668]	[588,271-723,382]

Absolute effect (S.D.)	5,701 (968)	199,543 (33,878)
95% CI	[3,713-7,573]	[129,956-265,067]

Relative effect (S.D.)	31% (5.2%)	31% (5.2%)
95% CI	[20%-41%]	[20%, 41%]

S.D. = Prior standard deviation of the Gaussian random walk of the local level.		
Posterior tail-area probability *p* (α=0.05): 0.00105		
Posterior probability of a causal effect: 99.89529%		

Table 3 is essential to know the dimensions of the “Trump Effect” according to the causal impact method in the areas of interpretation, prediction, tendency and contrast. The interpretation and predictive capacity of the model is obtained from the analysis of the post-intervention period during which the variable DJIA had an average value of 24,381 points and a standard deviation equal to 968. That is to say, if the intervention is ignored, a response would have been obtained of 18,680 points.

The “Trump Effect”, properly speaking, is measured by deducting the counterfactual prediction from the observed response (or reaction), considering it as a forecasted estimate of the causal effect of the intervention on the response variable: in this case, the effect is equal to an increment of 5,701 points in the DJIA index. By proposing as preliminary hypothesis a linear growth of the response variable during the post-intervention period, a constant trend in the growth of the DJIA is ascertained, equivalent to the non-negligible sum of 162.82 points per month (1,954.62 in annual terms).

Two of the parameters which define this methodology -the response in the absence of intervention and the effect of the intervention in absolute terms- can be statistically contrasted by constructing two confidence intervals at 95% level, as shown in Table 3. It can be observed that both predictors belong to the confidence intervals [16,808-20,668] and [3,713-7,573], respectively.

Using the same criteria, the cumulative procedure is applied by aggregating the individual points throughout the post-intervention data range. Accordingly, the response variable has an overall value of 853,338 and, ignoring the intervention, it should have an expected additional reaction of 653,795, with a 95% confidence interval for this prediction equal to [588.27K-723.38K]. Through the use of either the average or the cumulative approach, the DJIA as the response variable shows a strong average increase in relative terms of +31%, corresponding to a 95% prediction confidence interval [+20%,+40%].

In any case, it should be noted that the magnitude of the positive impacts observed during the intervention period is statistically significant and is unlikely to be due to purely random variations, since the probability of this effect occurring by mere chance is quite small (Bayesian one-sided tail-area probability p=0.001, see, e.g., Marsman and Wagenmakers, 2016).

**Figure 4:**
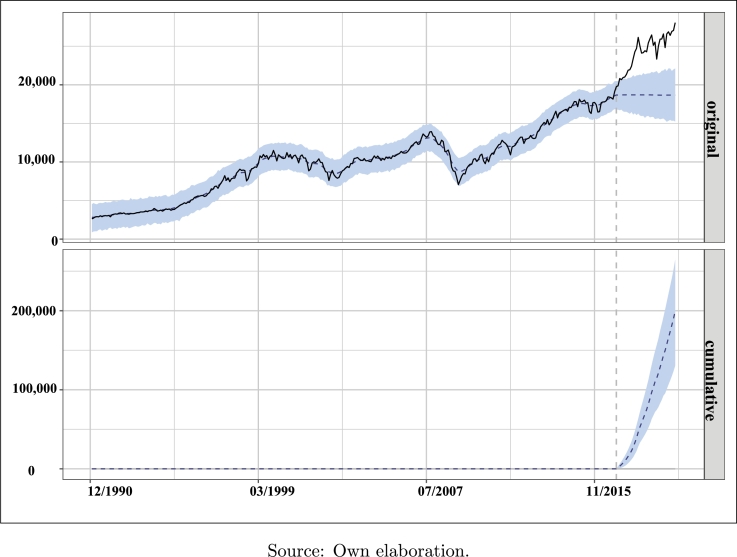
“Trump Effect”: causal impact of BCI on DJIA (I).

Fig. 4 represents the analysis of the “Trump Effect” taking into account the original (or average) and the cumulative approaches: the intervention date is indicated and, starting from this, the causal impact of the arrival of the Trump Administration on the performance of the DJIA index can be represented according to the previously defined confidence intervals.

**Figure 5:**
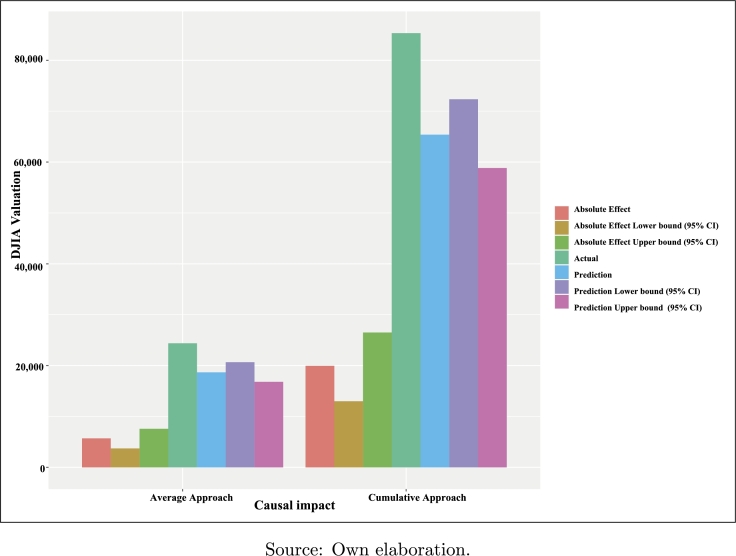
“Trump Effect”: causal impact of BCI on DJIA (II).

On the other hand, Fig. 5 also examines the same effect, but from a different perspective, by using a group bar plot to reflect the two types of results achieved in Table 3 (“absolute effect” and “prediction”) with respect to the average and cumulative approaches (the latter rescaled to base 10, in order to facilitate the comparison of both measures in the figure). In this multiple comparison of the value of parameters arising from the application of the causal impact methodology, the subsequent estimates of the DJIA index have been considered: average current value, absolute effect, and prediction (specifying, in the last two cases, the lower and upper bounds of each defined confidence interval).

## 5 Discussion

Among the main aspects which can be discussed in this paper, two in particular can be highlighted. First, we consider as symptomatic the causal relationship detected in the Granger sense between the variables DJIA and BCI. Second, it is even more significant to note that this causal relationship is fully supported by the causal-impact procedure (Brodersen et al., 2015), which quantifies it in absolute and relative terms.

This novel research represents a “third way” to add to others which have analyzed the “Trump Effect” employing Google Trends or Twitter (see, e.g., Born et al., 2017, Colonescu, 2018, Benton and Philips, 2019, de Area Leão Pereira et al., 2018, Brans and Scholtens, 2020, Klaus and Koser, 2020) or which have opted for a different database (see, e.g., Cox and Griffith, 2019, Selmi et al., 2020). Nevertheless, our empirical results are in all cases quite similar, showing a “reaction” of financial markets to the announcement of the future economic policies derived from the Trump Administration. One can clearly identify a “Trump Effect” included within the calendar effects (“US presidential cycle”) which, in any case, transgresses the fundamental bases of the EMH and which could be alternatively explained by any of the three hypotheses prevailing in the literature: OH, UIH and AMH. Although the EMH is controversial with both detractors and followers, it is still far from being ignored, even more so when it comes to hypothesize about the formation of financial bubbles (see, e.g., Chernomas and Hudson, 2017).

Born et al. (2017) used a single index model to estimate the systematic risk coefficient of 10 companies listed in the S&P500 index to conclude that irrational investors or “noise traders”, in the sense of Black (1986), were responsible for the rise in US financial asset prices. In our opinion, the results obtained from the application of the Bayesian causal-impact analysis (Brodersen et al., 2015) to determine the extent of the “Trump Effect” also lead to derive the presence of this kind of investor (irrational or uninformed) influenced by two factors: the uncertainty of the result of the US presidential election in 2016 and, in particular, the future impact of the Trump Administration's economic policies on US stocks exchanges (Wagner et al., 2018a, Wagner et al., 2018b).

Whether using the Volfefe Index to measure the impact of the “Trump Effect” on the European financial markets (Klaus and Koser, 2020), the number of tweets (Brans and Scholtens, 2020) or the exact appearance of the term “Donald Trump” in Google Trends (de Area Leão Pereira et al., 2018), the “Trump Effect” has a significant (positive) impact on the performance of the analyzed markets (not exclusively the US financial markets), which, unlike other works, has been able to be quantified. This is the main contribution of this paper.

## 6 Conclusions

One of the current challenges of Behavioral and Experimental Finance is to show empirically the existence of the so-called “Calendar Effects”. In this research work, by using an almost unprecedented methodology in the field of finance, it has been empirically demonstrated, in line with Hirsch and Hirsch (2010), that the latest US presidency exhibits one of these effects (United States presidential election cycle), caused by the doubts and uncertainties among investors related to the economic policy measures which the new administration will take and, to what extent it will carry out its electoral promises particularly focused on fiscal, labor and foreign trade policies (Wagner et al., 2017, Wagner et al., 2018a, Wagner et al., 2018b).

It is believed that this study can offer substantial improvements over the existing literature for three reasons; first, a very appropriate and flexible methodology has been implemented to analyze the United States presidential election cycle; second, it allows for the generation of numerous case studies in which other presidential changes can be studied; and finally, since we start from an objective, quantifiable and predictable causal relationship, the inconsistency of the EMH as to the randomness of stock prices can be demonstrated from an empirical basis.

In addition, the following policy implications can be observed. As Benton and Philips (2019) stated, any new information related to the probable political direction which the new presidential administration may adopt has a direct impact on the stability of financial markets, inducing a higher volatility and uncertainty if the winning candidate turns out to be unexpected (Cox and Griffith, 2019). It also produces a significant variation in short-term exchange rates (see, e.g., Colonescu, 2018, Benton and Philips, 2019). This fact may be due to the new foreign policy guidelines sponsored by the Trump Administration. Likewise, according to the empirical approach proposed by Selmi et al. (2020), the uncertainties concerning monetary-economic policy (basically, those indicated above) would be transferred not only to financial markets but to several key macroeconomic dimensions, generating possible inflationary movements.

This research offers support to investors in several areas; for instance, by establishing a suitable pre-intervention period, the Political Year Cycle Strategy (PYCS, see Nickles and Valadez, 2009) can be supported by reducing the time exposure to financial markets by investing in the DJIA 1 year out of 4 and remaining in risk-free assets (i.e., commercial paper rates or a money market) for the other 3 years, by assuming that the investment is made only during the year before the US presidential election.

Another alternative investment strategy in which the procedure of Brodersen et al. (2015) can be employed within the scope of the “Calendar Effects” would involve calculating the average variations in the DJIA in each of the United States' presidencies, starting from 1896, the date of creation of this index (Grover Cleveland presidency), by using the Business Confidence Index (BCI) as a variable *proxy*, or any other which can be considered representative of the level of investor confidence in the financial markets. In this sense, two pre-intervention and post-intervention periods must be defined for each of the presidencies depending on the period which the investor or analyst considers appropriate (for example, taking as an intervention date the US presidential inauguration day). Thus, the average of the presidential effects to date should serve as a representative measure for the financial analyst in the assessment of the “US presidential cycle” according to the methodology employed in this work.

## Declarations

### Author contribution statement

S. Cruz Rambaud: Contributed reagents, materials, analysis tools or data; Wrote the paper. P.A. Martín Cervantes: Conceived and designed the experiments; Performed the experiments; Analyzed and interpreted the data; Wrote the paper.

### Funding statement

This research has been funded by the Spanish 10.13039/501100003329Ministry of Economy and Competitiveness, grant number DER2016-76053R.

### Competing interest statement

The authors declare no conflict of interest.

### Additional information

No additional information is available for this paper.

## Data Availability

To carry out this research work, data from the following sources have been utilized: OECD: Business confidence index (BCI), 2019, https://www.oecd-ilibrary.org/economics/business-confidence-index-bci/indicator/english_3092dc4f-en. Bloomberg: Bloomberg Professional Services, 2019, https://www.bloomberg.com/professional/solution/financial-data-management/.
